# Predictors of seizures after endovascular aneurysm exclusion: an updated systematic review and meta-analysis

**DOI:** 10.3389/fneur.2026.1869671

**Published:** 2026-08-28

**Authors:** Muhammad Aftab Hassan, Maryam Amjad, Shifa Fatima, Jannat Yaqoob, Abdul Rahim Soroush, Sibtain Ali, Antonia Malli

**Affiliations:** 1 Department of Neurosurgery, Wah Medical College, Wah Cantt, Punjab, Pakistan; 2 Department of Medicine, Wah Medical College, Wah Cantt, Punjab, Pakistan; 3 Department of Medical Sciences, Wah Medical College, Wah Cantt, Punjab, Pakistan; 4 Department of Medical Sciences, Shahida Islam Medical College, Lodhran, Punjab, Pakistan; 5 Medical Faculty, Department of Medical Sciences, Balkh University, Mazar-i-Sharif, Afghanistan; 6 Department of Medicine, Baqai Medical University, Karachi, Sindh, Pakistan; 7 Southmead Hospital, North Bristol NHS Trust, Department of Neurosurgery, Bristol, United Kingdom

**Keywords:** endovascular treatment, fisher grade, hydrocephalus, intracranial aneurysm, seizures

## Abstract

Endovascular aneurysm exclusion has revolutionized neurovascular care, yet postoperative seizures remain a significant cause of morbidity. As endovascular technologies evolve from simple coiling to complex flow diversion, identifying factors associated with postoperative seizures is essential for improving risk stratification and neurocritical care. This systematic review and meta-analysis, registered in PROSPERO (CRD420261375370), followed PRISMA 2020 guidelines. A comprehensive search of MEDLINE, Scopus, Embase, and the Cochrane Library was conducted through April 13, 2026. Studies published since 2016 investigating factors associated with seizures after endovascular treatment (coiling, flow diversion, or stent-assisted techniques) were included. Random-effects meta-analysis was performed using R to calculate pooled odds ratios (OR) and 95% confidence intervals (CI), with subgroup and sensitivity analyses performed where feasible. Six studies met the inclusion criteria for qualitative and quantitative synthesis. Radiological markers showed the strongest associations with post-procedural seizures. Hydrocephalus demonstrated the highest association (OR 3.91, 95% CI: 1.22 to 12.53), followed by Fisher grade 3 + 4 blood distribution (OR 3.29, 95% CI: 1.55 to 6.97, *I*^2^ = 0%). Regarding clinical grading, Hunt-Hess grade 1 to 3 was significantly associated with seizure occurrence (OR 3.15, 95% CI: 1.73 to 5.72), whereas WFNS grade 4 to 5 was also associated with seizure occurrence (OR 1.86, 95% CI: 1.14 to 3.02), although heterogeneity was moderate (*I*^2^ = 60.2%). Factors such as age, sex, MCA involvement, and cerebral oedema did not reach statistical significance (*p* > 0.05). Quality assessment using the Newcastle–Ottawa Scale (NOS) indicated overall moderate methodological quality, although the evidence was limited by observational study designs, heterogeneous seizure outcome definitions, and a small number of included studies. Radiological severity of the initial hemorrhage, particularly dense subarachnoid blood and secondary cerebrospinal fluid disturbances, was associated with a higher likelihood of postoperative seizures, whereas anatomical location and patient demographics showed no significant associations. These findings should be interpreted cautiously and may assist future risk stratification; however, they do not alone support specific antiepileptic prophylaxis strategies. Further prospective studies incorporating standardized seizure definitions and continuous EEG monitoring are warranted.

**Systematic review registration:**

https://www.crd.york.ac.uk/prospero/search, identifier CRD420261375370.

## Introduction

Intracranial aneurysms represent a critical neurovascular challenge, with their rupture leading to aneurysmal subarachnoid haemorrhage (aSAH), a condition characterized by a 30-day mortality rate exceeding 30% and profound long-term disability (1). Over the last two decades, the management of these lesions has undergone a tectonic shift from microsurgical clipping toward endovascular aneurysm exclusion (1, 2). While endovascular techniques ranging from simple coil embolization to advanced flow diversion offer a less invasive alternative with reduced perioperative morbidity, the emergence of postoperative seizures and new-onset epilepsy remains a persistent and poorly understood clinical burden (3, 4). The incidence of seizures following endovascular intervention is not negligible; classic longitudinal data from the International Subarachnoid Aneurysm Trial identified a seizure frequency of approximately 8% for patients treated with endovascular coiling, compared to 14% for those undergoing microsurgical clipping (4, 5). Recent evidence suggests that the cumulative incidence of seizures in stroke patients following endovascular treatment is approximately 5.8%, though this rate can escalate significantly depending on the clinical presentation and injury severity (6). In specific cohorts of unruptured aneurysms, the risk of developing seizures following intervention has been reported as high as 15.7% in historical series, highlighting that even elective “low-risk” exclusions are not immune to epileptogenic complications (7).

Identifying reliable factors associated with post-endovascular seizures is paramount for clinical risk stratification and may help inform future studies evaluating prophylactic antiepileptic drugs (4, 8). Current research indicates that clinical severity at presentation, as measured by the World Federation of Neurosurgical Societies or Hunt and Hess scales, is a dominant predictor; specifically, high-grade patients (WFNS IV–V) face a 3.34-fold increase in seizure risk (OR 3.34, 95% CI: 1.15–9.73) compared to lower-grade counterparts. Furthermore, radiological markers such as the presence of severe cerebral vasospasm, intracranial infarction, and significant cerebral oedema have been consistently identified as triggers for post-procedural cortical irritability (8, 9). Anatomical location also plays a definitive role, with middle cerebral artery aneurysms associated with a higher seizure threshold due to their proximity to the eloquent cortex (3, 5).

Despite these insights, a significant “predictive gap” remains. The rapid evolution of endovascular hardware including flow diverters, flow-disruptor devices, and newer-generation embolic agents has introduced unique hemodynamic and biological variables that may influence epileptogenesis in ways not captured by earlier meta-analyses (1, 4). Moreover, existing literature presents conflicting data regarding the protective versus provocative effects of different endovascular modalities (5, 9). This updated systematic review and meta-analysis aim to synthesize the most recent high-level evidence to delineate the clinical, anatomical, and procedure-related predictors of seizures after endovascular aneurysm exclusion, thereby providing a robust evidence-based framework for personalized neurocritical care and seizure prophylaxis.

## Materials and methods

This systematic review and meta-analysis were conducted in strict adherence to the Preferred Reporting Items for Systematic Reviews and Meta-analyses (PRISMA) 2020 guidelines. The protocol was prospectively registered in the PROSPERO international database of systematic reviews CRD420261375370. Eligibility criteria were defined using the population, intervention, comparison, and outcome framework. The population (P) includes patients of any age undergoing endovascular exclusion of intracranial aneurysms, including techniques such as coiling, flow diversion, or stent-assisted procedures. The intervention (I) focuses on potential predictors of post-procedural seizures, such as aneurysm size, location (for example, involvement of the middle cerebral artery), rupture status, and the specific endovascular modality used. The comparison (C) is between patients who developed seizures and those who remained seizure-free. The outcomes (O) included early postoperative seizures, late postoperative seizures, in-hospital seizures, epilepsy at 12 months, and overall postoperative seizure occurrence, as defined by the individual included studies, along with factors evaluated for their association with seizure occurrence, including Hunt Hess grade 1–3, age, sex, comorbidity (including hypertension), WFNS grade 4–5, Fisher grade 3 + 4, middle cerebral artery involvement, cerebral oedema, and hydrocephalus Table 1.

**Table 1:** PICO framework.

Element	Component	Description
P	Population	Patients of any age undergoing endovascular exclusion of intracranial aneurysms (including coiling, flow diversion, or stent-assisted techniques).
I	Intervention	Potential predictors of post-procedural seizures: Aneurysm size and location (e.g., middle cerebral artery). Rupture status. Specific endovascular modalities.
C	Comparison	Patients who developed seizures versus those who remained seizure-free.
O	Outcomes	Seizure events: Early seizure, late seizure, in-hospital seizure, any seizure, and epilepsy at 12 months. Predictor variables: Hunt-Hess grade 1–3, WFNS grade 4–5, Fisher grade 3 + 4. MCA involvement, cerebral oedema, and hydrocephalus. Age, sex, and comorbidity (hypertension).

To ensure contemporary and relevant data, we included English-language articles published since 2016 featuring non-randomized designs, including prospective and retrospective comparative cohorts and propensity-matched analyses. All studies were required to report clearly defined postoperative seizure outcomes (e.g., early, late, in-hospital, or overall seizure occurrence, as defined by the individual studies) stratified by factors evaluated for their association with seizure occurrence to allow for qualitative and quantitative synthesis. The search strategy systematically incorporated Medical Subject Headings terms and keywords pertinent to the PICO elements, including “intracranial aneurysm,” “endovascular treatment,” “seizures,” “epilepsy,” “coiling,” and “flow diversion.” The literature search was conducted on April 13, 2026, across four major databases: MEDLINE, Scopus, Embase and the Cochrane Library. The initial database search identified 7,883 records. After removing duplicates, 7,765 titles and abstracts were screened. This first stage excluded 7,722 articles due to irrelevance, leaving 41 full-text articles for detailed evaluation. The full-text review resulted in 35 further exclusions, with 6 studies ultimately meeting all inclusion criteria for the qualitative synthesis Table 2.

**Table 2:** Search information by database (PRISMA-S item 7).

Database/Source	Platform/Interface	Full search strategy/Query	Date last searched	Time span	Filter/Limit applied	Records retrieved
MEDLINE	PubMed	(((“Intracranial Aneurysm”[MeSH] OR “Cerebral Aneurysm”[tiab] OR “Intracranial Aneurysm”[tiab] OR “Subarachnoid Hemorrhage”[MeSH] OR “Subarachnoid Hemorrhage”[tiab])) AND (“Endovascular Procedures”[MeSH] OR “Endovascular”[tiab] OR “Coiling”[tiab] OR “Embolization, Therapeutic”[MeSH] OR “Embolization”[tiab] OR “Flow Diverter”[tiab] OR “Intrasaccular device”[tiab])) AND (“Seizures”[MeSH] OR “Epilepsy”[MeSH] OR “Seizures”[tiab] OR “Epilepsy”[tiab] OR “Convulsions”[tiab] OR “Epileptogenesis”[tiab]) AND (“Risk Factors”[MeSH] OR “Incidence”[MeSH] OR “Risk Factors”[tiab] OR “Predictors”[tiab] OR “Association”[tiab] OR “Incidence”[tiab])	13 April 2026	Up to April 2026	Filters: English, humans, adult: 19 + years	40
Embase	Ovid	(((intracranial AND “aneurysm”/mj AND endovascular AND “therapy”/mj AND “coiling”/mj OR flow) AND diverter AND postoperative AND seizure OR postprocedural) AND seizure AND risk AND factor OR predictor) AND ([adult]/lim OR [aged]/lim OR [very elderly]/lim) AND ([embase]/lim OR “clinical trial”:dtype) AND ([controlled clinical trial]/lim OR [randomized controlled trial]/lim) AND [2016–2026]/py AND [humans]/lim AND [english]/lim AND [abstracts]/lim AND [clinical study]/lim	13 April 2026	2016–2026	Filters: English, humans, adult: 18 + years, randomized controlled trial, controlled clinical trial, Abstracts, embase	7,484
Scopus	Elsevier	(Intracranial aneurysm OR cerebral aneurysm) AND endovascular AND (coiling OR flow diverter OR embolization) AND (postoperative seizure OR postprocedural seizure) AND (risk factor OR predictors) AND (LIMIT-TO (DOCTYPE, “ar”)) AND (LIMIT-TO (EXACTKEYWORD, “Human”)) AND (LIMIT-TO (LANGUAGE, “English”))	13 April 2026	Up to April 2026	Human, English, article	351
Cochrane library	Wiley online	(Intracranial aneurysm OR cerebral aneurysm OR aneurysm) AND (endovascular OR coiling OR embolization OR flow diverter) AND (seizure OR epilepsy) AND (postoperative OR postprocedural)	13 April 2026	13 April 2026	Only trials	8

Study selection was performed by two independent reviewers, MH and MA, who initially screened titles and abstracts followed by a comprehensive full-text evaluation. Any discrepancies were resolved through discussion or by consultation with a third reviewer, SQ. Articles were excluded if they lacked appropriate comparison groups, did not provide extractable seizure data, or were non-human studies. Data extraction was performed using a pre-piloted, standardized data sheet, capturing author details, study design, patient demographics, aneurysm characteristics (size, location, morphology), procedural specifics, and the following outcomes: early postoperative seizure, late postoperative seizure, in-hospital seizure, epilepsy at 12 months, and overall postoperative seizure occurrence, as defined by the individual included studies, together with factors evaluated for their association with seizure occurrence, including Hunt Hess grade 1–3, age, sex, comorbidity (hypertension), WFNS grade 4–5, Fisher grade 3 + 4, MCA involvement, cerebral edema, and hydrocephalus, with corresponding odds ratios and confidence intervals. This process was carried out independently by reviewers MH and MA, with disagreements resolved by reviewer SQ. The authors did not contact the study authors for missing data. The review relied strictly on the data provided in the published literature Table 3.

**Table 3:** Characteristics of core studies included in meta-analysis.

Study	Population	Key finding
Bögli (10)	aSAH cohort	Status epilepticus significantly impacts outcomes.
Nakashima (13)	Endovascular	Refined predictors in the modern device era.
Peng (11)	Ruptured	Validated clinical and radiological grading.
Nathan (14)	Endovascular	Hunt-Hess severity and infarction as major risks.
Le (4)	aSAH	Identified hydrocephalus as a critical seizure trigger.
Baticulon (12)	aSAH	Established long-term epilepsy risk post-intervention.

The methodological quality of non-randomized studies was assessed using the Newcastle–Ottawa Scale (NOS). Studies were evaluated across the domains of selection, comparability, and outcome assessment, and were categorized according to their overall methodological quality based on NOS scores. Certainty of evidence was assessed using the Grading of Recommendations, Assessment, Development and Evaluation framework. Statistical analysis was conducted using R software. For dichotomous outcomes (seizure incidence), data were pooled using Mantel–Haenszel odds ratios with 95% confidence intervals. Continuous outcomes were pooled using inverse-variance weighted mean differences. A random-effects model was utilized to account for anticipated clinical and methodological heterogeneity. Pre-specified subgroup analyses were performed according to individual clinical and radiological factors, and sensitivity analyses were undertaken where feasible to evaluate the robustness of the pooled estimates. Statistical heterogeneity was assessed using the *I*^2^ statistic, with *I*^2^ > 50% considered substantial. Where meta-analysis was precluded by high heterogeneity or insufficient data, a narrative synthesis was prioritized to interpret the findings. The outcomes extracted for analysis were early postoperative seizure, late postoperative seizure, in-hospital seizure, epilepsy at 12 months, and overall postoperative seizure occurrence, as defined by the individual included studies, together with the factors evaluated for their association with seizure occurrence and their corresponding odds ratios and confidence intervals.

## Quality assessment

The quality assessment, conducted using the Newcastle-Ottawa Scale (NOS), demonstrated variability in the methodological quality. For the cohort studies, the NOS was utilized to evaluate selection, comparability, and outcome/exposure assessment.

Two studies, Bögli et al. (10) and Peng et al., (11) demonstrated high methodological quality, achieving NOS scores of 9 and 8 stars, respectively. Conversely, Baticulon et al., (12) Le et al., (4) and Nakashima et al. (13) Demonstrated moderate methodological quality, with scores of 7, 6, and 7 stars, respectively, whereas Nathan et al. (14) Demonstrated comparatively lower methodological quality, with a total score of 5 stars.

A domain-level analysis suggested that the comparability domain represented the most frequent methodological limitation across the included studies. Specifically, Nakashima et al. (13) and Nathan et al. (14) Showed deficiencies in controlling for confounding variables, achieving only 1 star in the comparability domain. Furthermore, lower scores in the outcome/exposure assessment domain were observed in studies such as Le et al. (4) and Nathan et al. (14) (1 star each), reflecting potential limitations in follow-up and outcome assessment.

**Figure 1:**
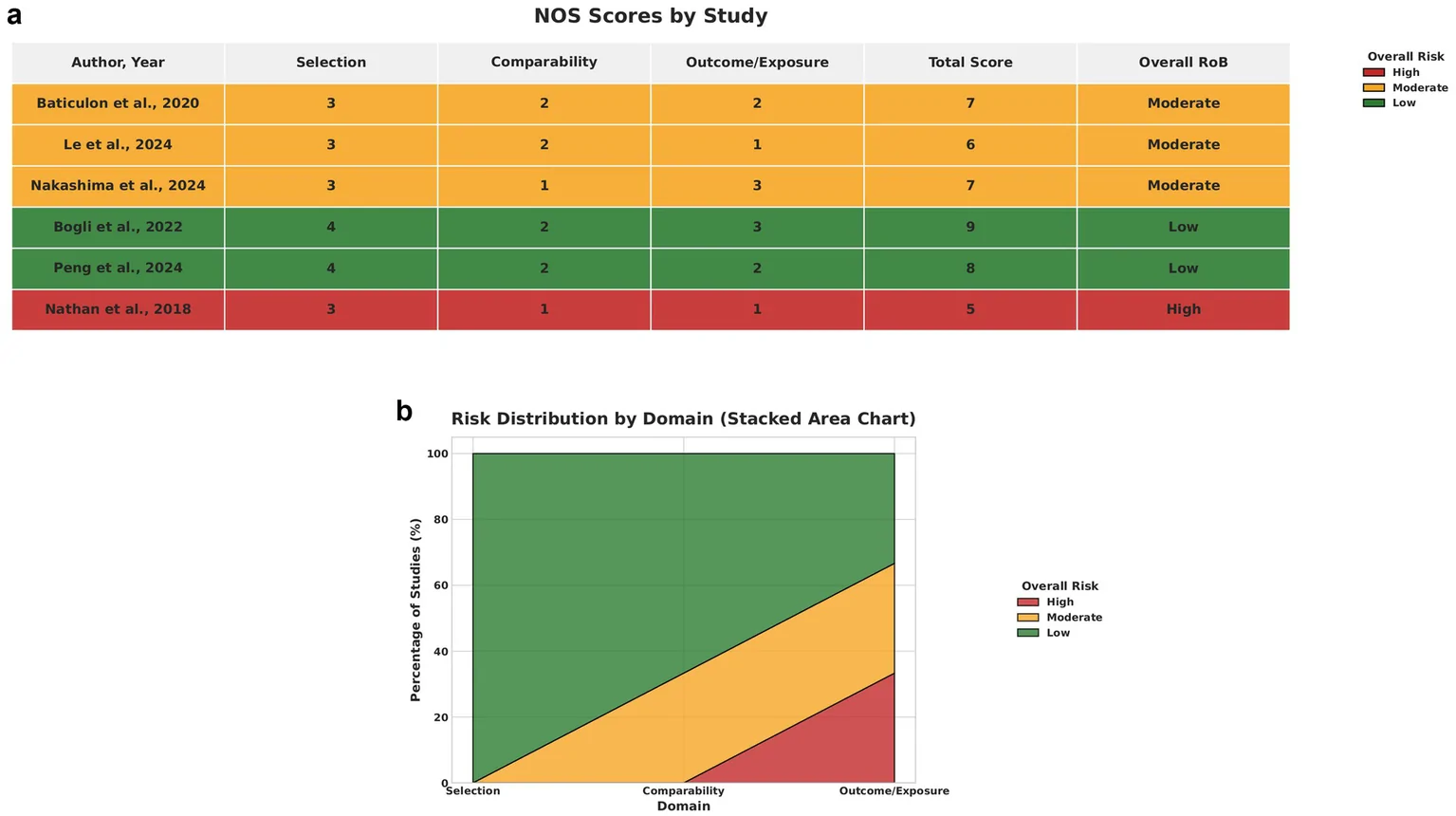
**(a,b)** ROB assessment.

In contrast, the selection domain generally demonstrated stronger methodological quality across the included studies. The selection domain for the cohort studies was the most robust, with Bögli et al. (10) and Peng et al. (11) Achieving maximum points (4 stars). Despite variations in NOS scores, most studies performed well in the selection domain, although methodological limitations in comparability and outcome assessment should be considered when interpreting the pooled findings Figure 1.

## Results

**Figure 2:**
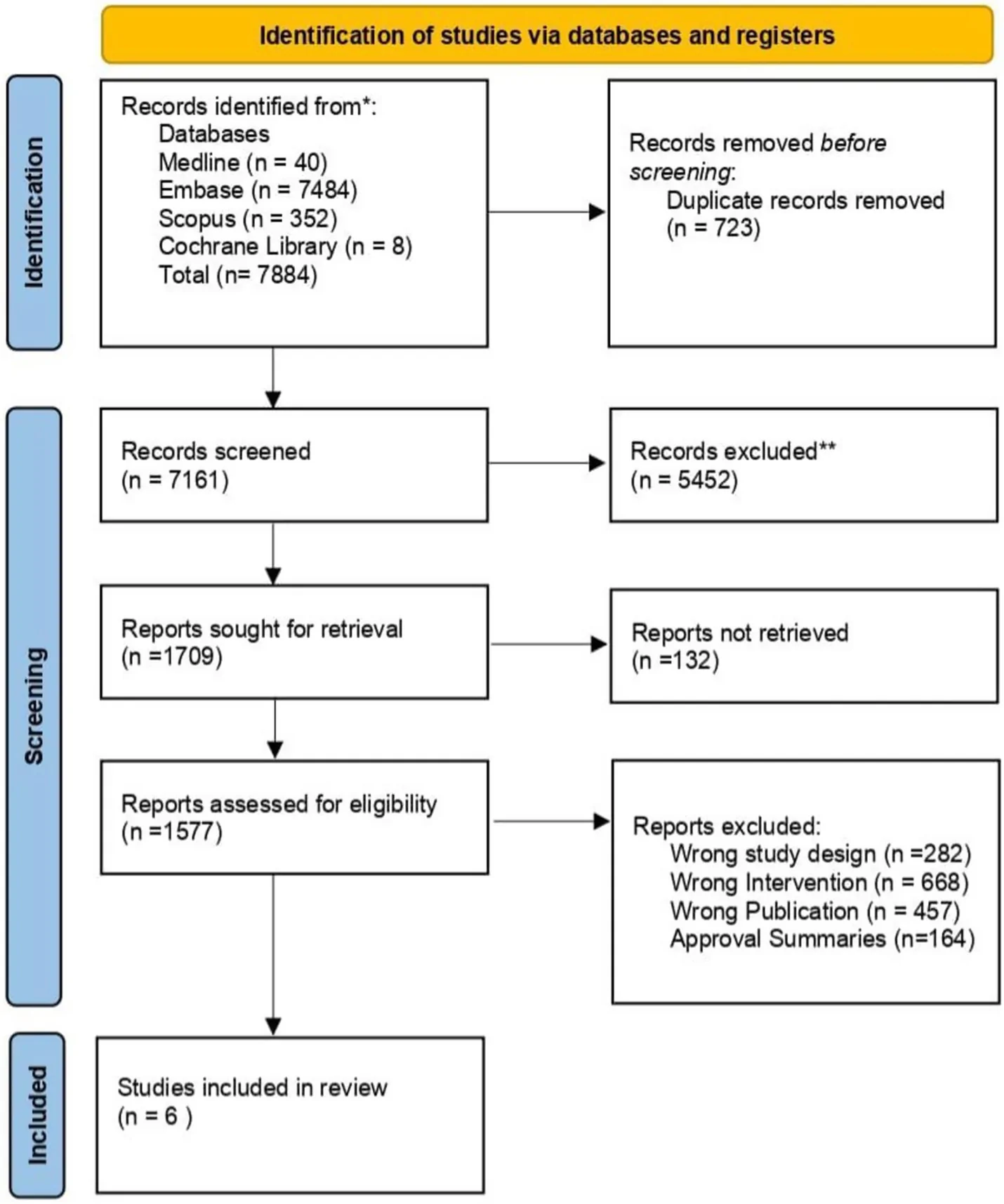
PRISMA diagram.

Data were extracted from studies identified through the systematic search. The initial database search identified 7,883 records. After removing duplicates, 7,765 titles and abstracts were screened. This first stage excluded 7,722 articles due to irrelevance, leaving 41 full-text articles for detailed evaluation. The full-text review resulted in 35 further exclusions, with 6 studies ultimately meeting all inclusion criteria for the qualitative synthesis. Adequate quantitative data were available for random-effects meta-analysis of Pre-specified clinical and radiological factors associated with postoperative seizure occurrence, with subgroup and sensitivity analyses performed where feasible. Findings from the narrative synthesis were interpreted alongside the meta-analysis results with consideration of methodological heterogeneity, variability in seizure outcome definitions, and the observational nature of the included studies Figure 2.

### Meta-analysis of seizure predictors

#### Hunt-Hess grade 1–3

**Figure 3:**
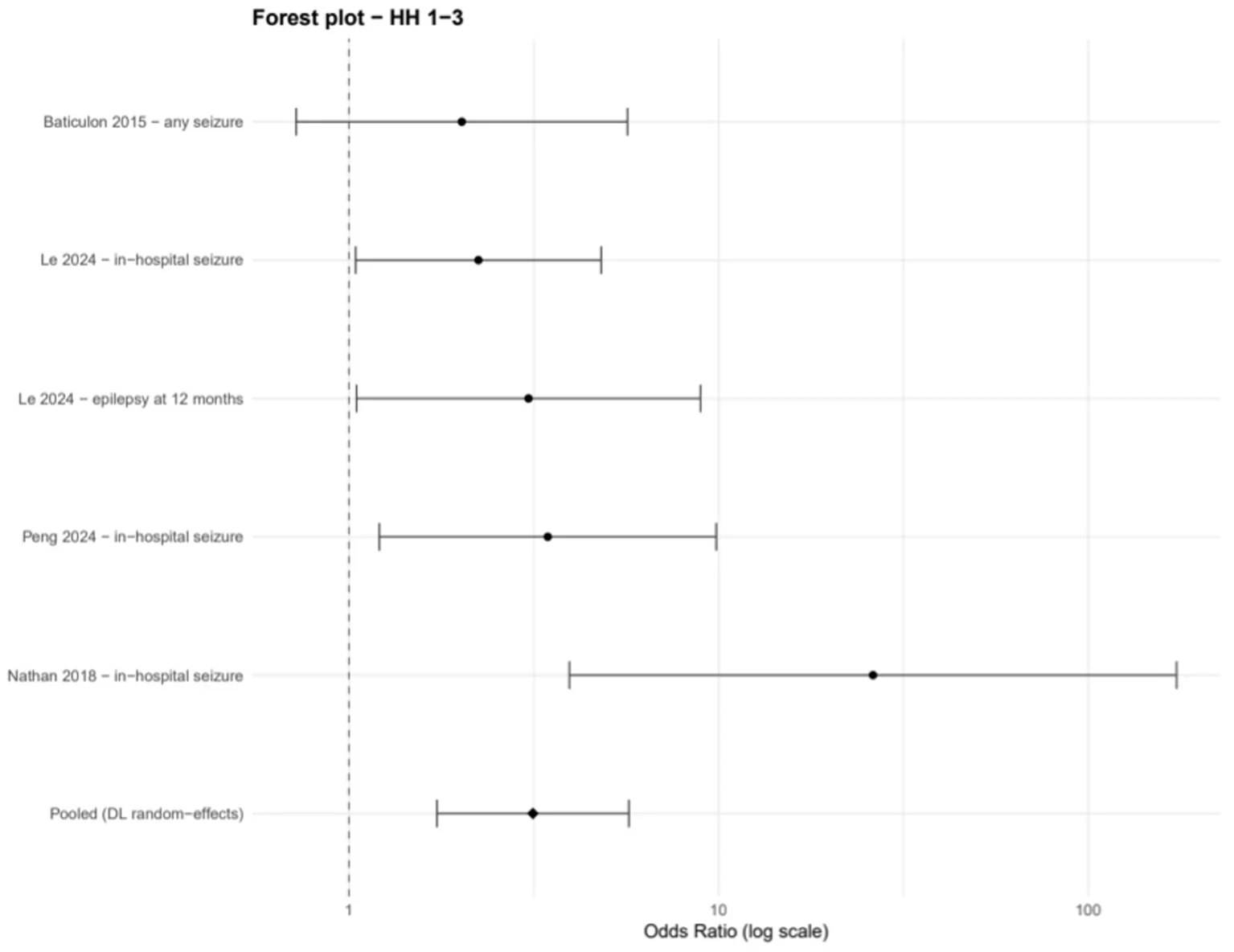
Forest plot for Hunt-Hess grade 1–3.

Among the evaluated clinical factors, Hunt-Hess grade 1–3 showed the strongest association with Post-aneurysmal subarachnoid hemorrhage seizures (Figure 3). Meta-analysis of five studies found a statistically significant pooled odds ratio of 3.15 (95% CI: 1.73–5.72, (*p* < 0.001)). The heterogeneity was moderate (*I*^2^ = 35.7%), suggesting generally consistent effect estimates across the included studies, although some between-study variability remained. Subgroup analysis of in-hospital seizures (three studies) demonstrated a pooled OR of 3.25 (95% CI: 1.81–5.85), consistent with the overall analysis. Single-study analyses of overall seizure occurrence and epilepsy at 12 months demonstrated directionally consistent associations; however, the wider confidence intervals reflect greater statistical uncertainty and should be interpreted cautiously.

#### Fisher grade 3 + 4

**Figure 4:**
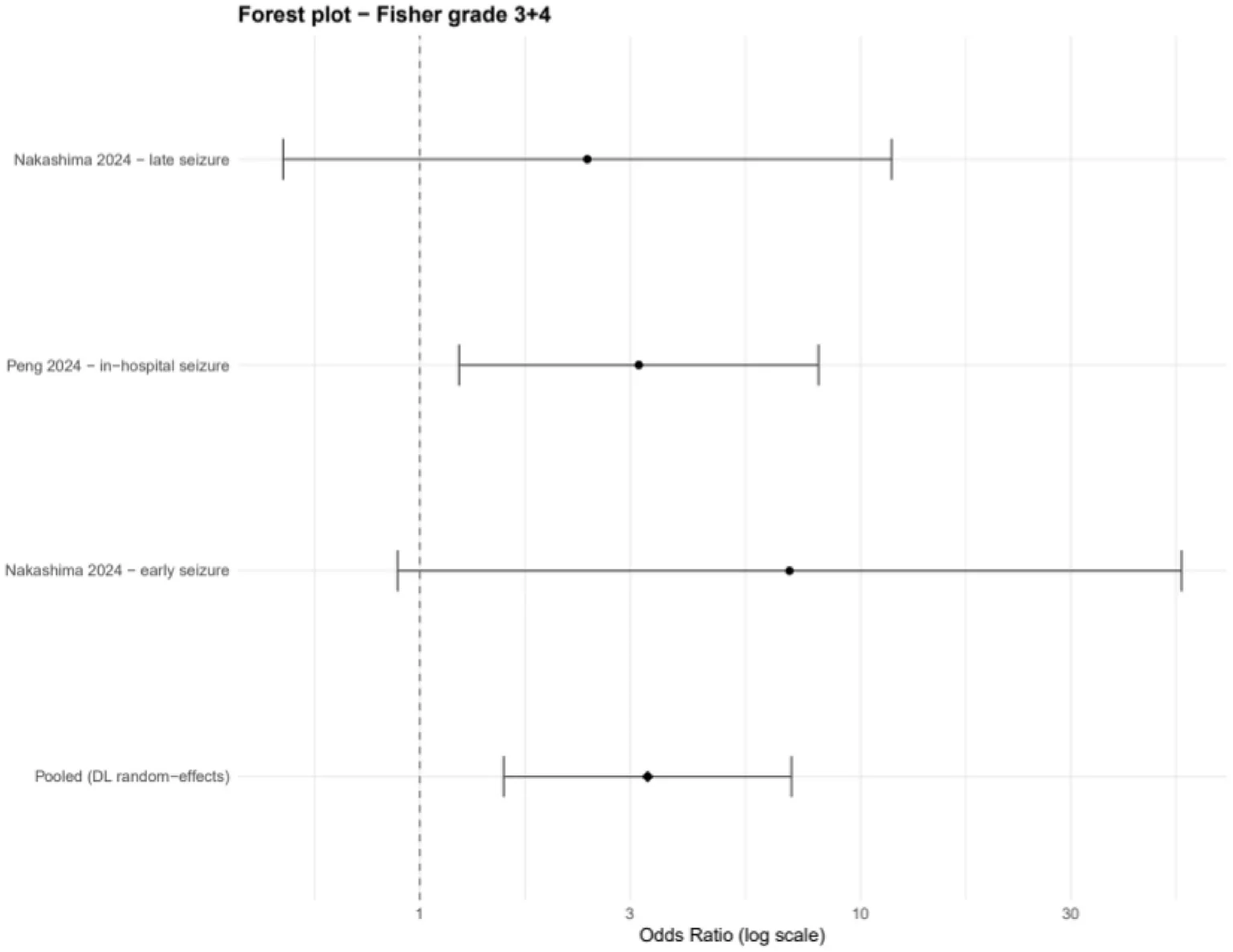
Forest plot for fisher grade 3 + 4.

Fisher grade 3 + 4 demonstrated the second-strongest association with Post-aneurysmal subarachnoid hemorrhage seizures, with a pooled OR of 3.29 (95% CI: 1.55–6.97; *p* = 0.002). Heterogeneity was negligible (*I*^2^ = 0%), indicating consistent effect estimates across the three included studies Figure 4.

#### WFNS grade 4–5

**Figure 5:**
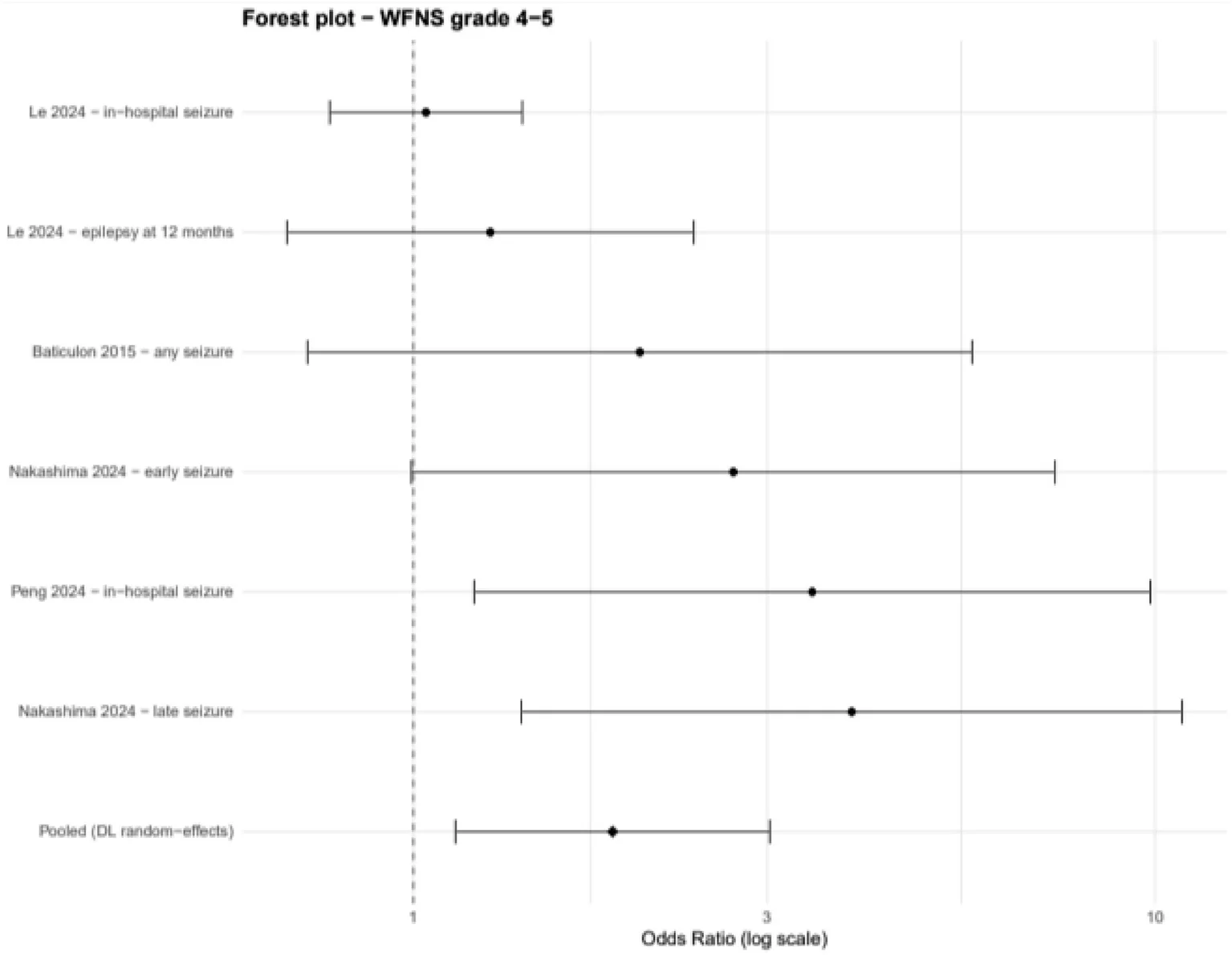
Forest plot for WFNS grade 4–5.

WFNS grade 4–5 was associated with Post-aneurysmal subarachnoid hemorrhage seizures across six studies, with a pooled OR of 1.86 (95% CI: 1.14–3.02; *p* = 0.013). However, substantial heterogeneity was observed (*I*^2^ = 60.2%), indicating variability in effect estimates across the included studies; therefore, this association should be interpreted cautiously Figure 5.

#### Hydrocephalus

**Figure 6:**
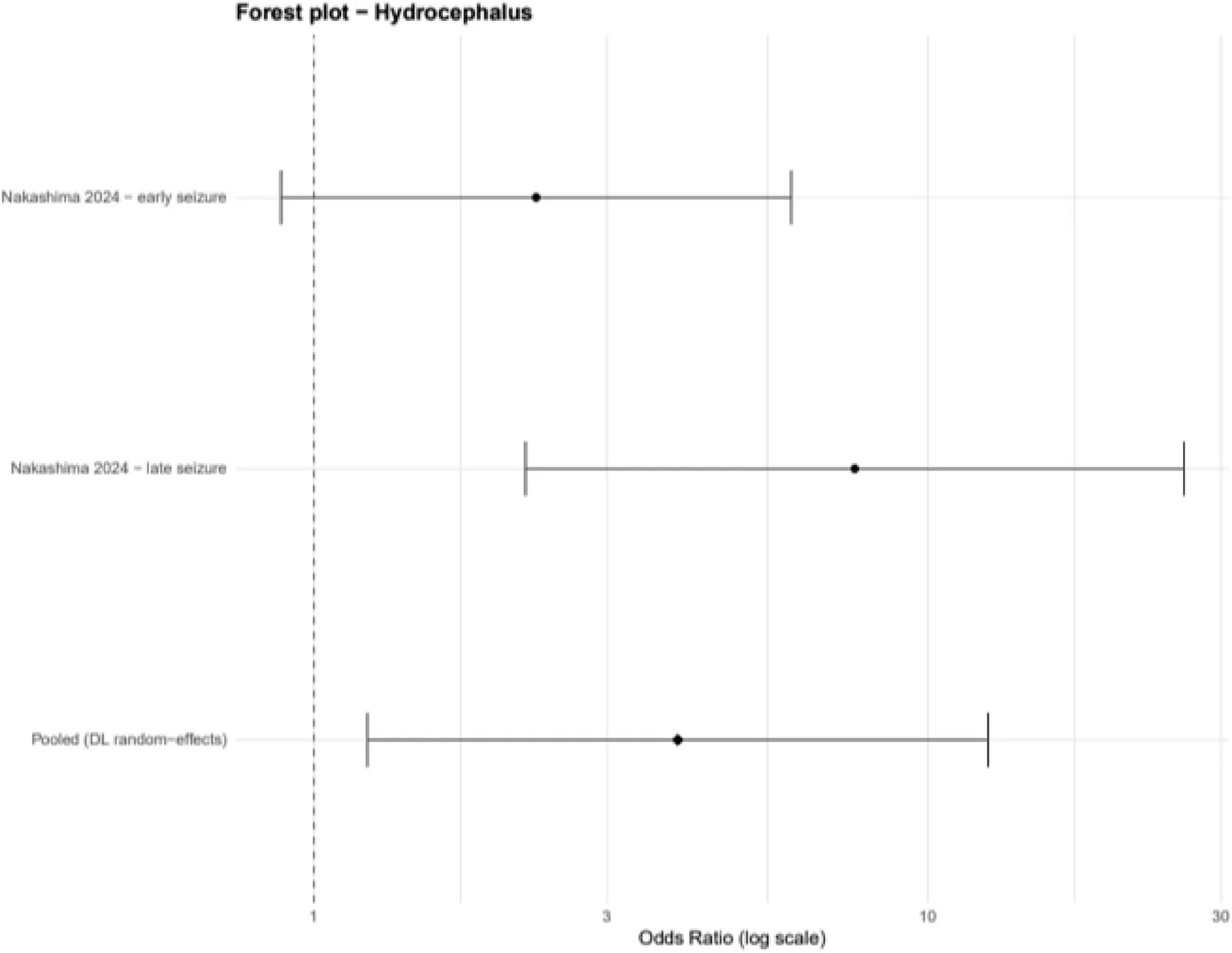
Forest plot for hydrocephalus.

Hydrocephalus was associated with Post-aneurysmal subarachnoid hemorrhage seizures in the two included studies, with a pooled OR of 3.91 (95% CI: 1.22–12.53; *p* = 0.022). However, the wide confidence interval, moderate heterogeneity (*I*^2^ = 55.5%), and the limited number of included studies warrant cautious interpretation of this association Figure 6.

### Non-significant predictors

#### Age

**Figure 7:**
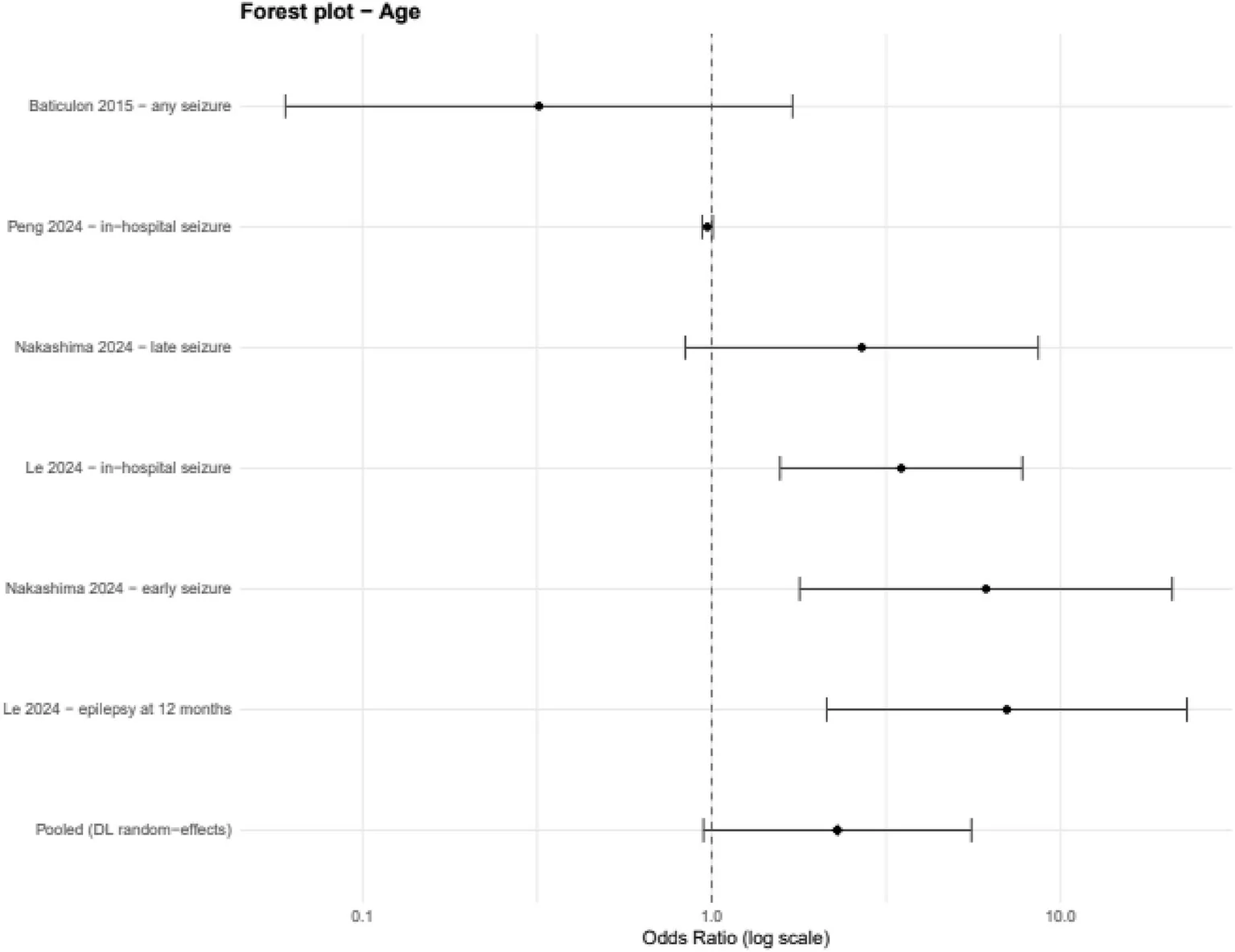
Forest plot for age.

There was a borderline association between age and post-aneurysmal subarachnoid hemorrhage seizures (pooled OR = 2.29, *p* = 0.066); however, the substantial heterogeneity observed (*I*^2^ = 85.1%, the highest among all evaluated factors) indicates considerable inconsistency across the included studies. This suggests that the studies were not in agreement regarding the association between age and post-aneurysmal subarachnoid hemorrhage seizures, or the magnitude of this association, highlighting variability in study populations, methodologies, seizure outcome definitions, and potential confounding factors. Accordingly, these findings should be interpreted cautiously Figure 7.

#### Comorbidity, MCA involvement, cerebral edema, and sex

**Figure 8:**
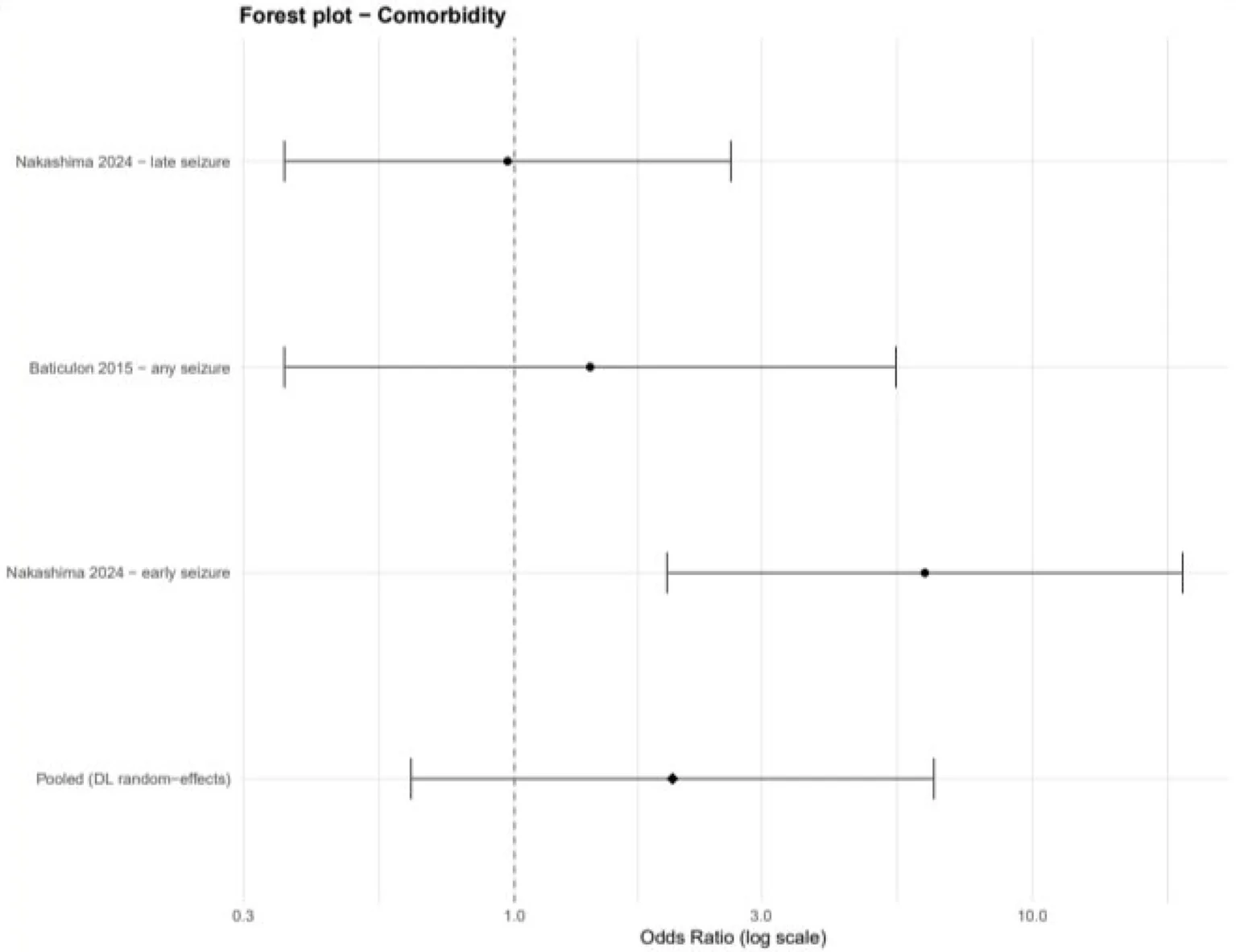
Forest plot for comorbidity.

**Figure 9:**
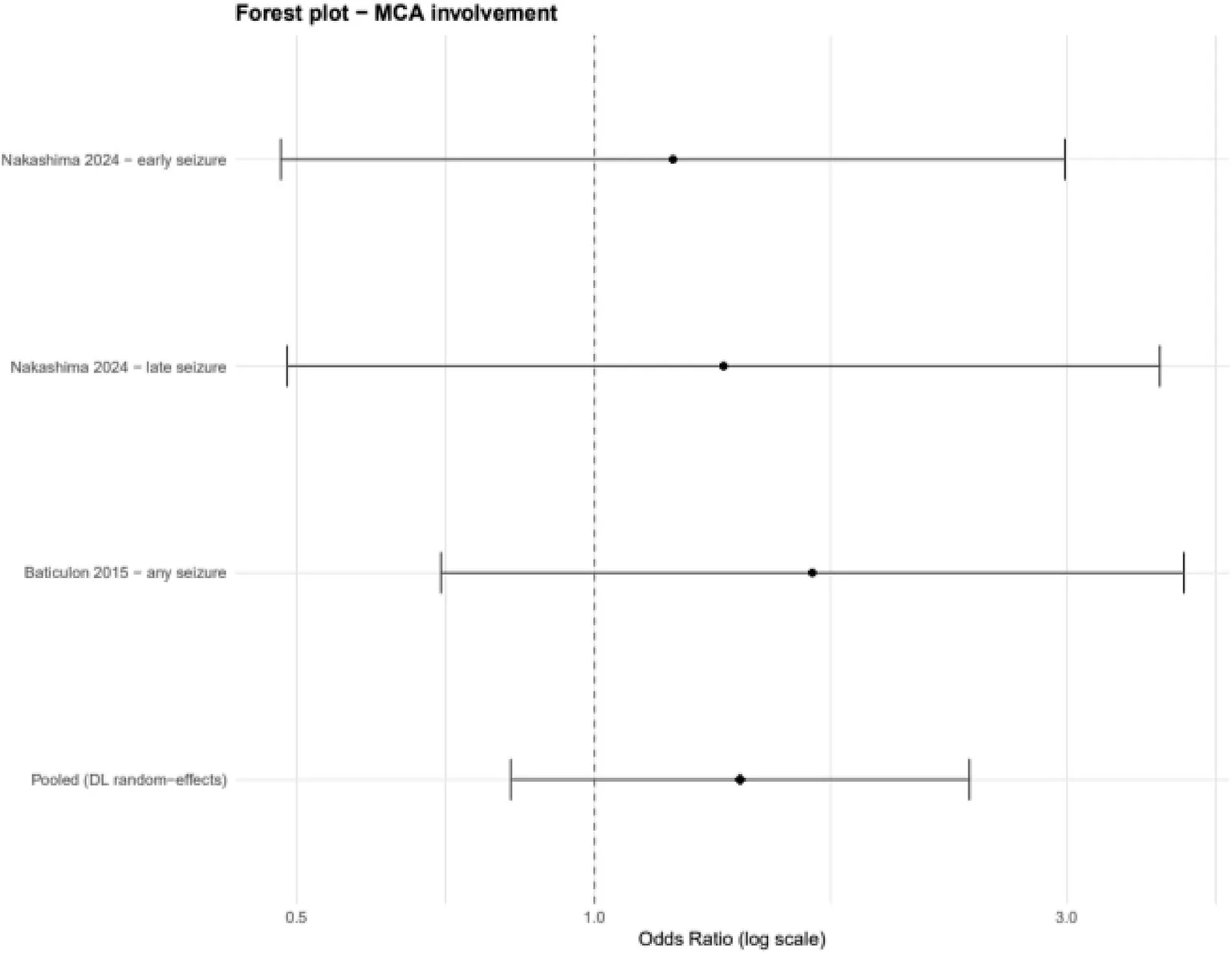
Forest plot for MCA involvement.

**Figure 10:**
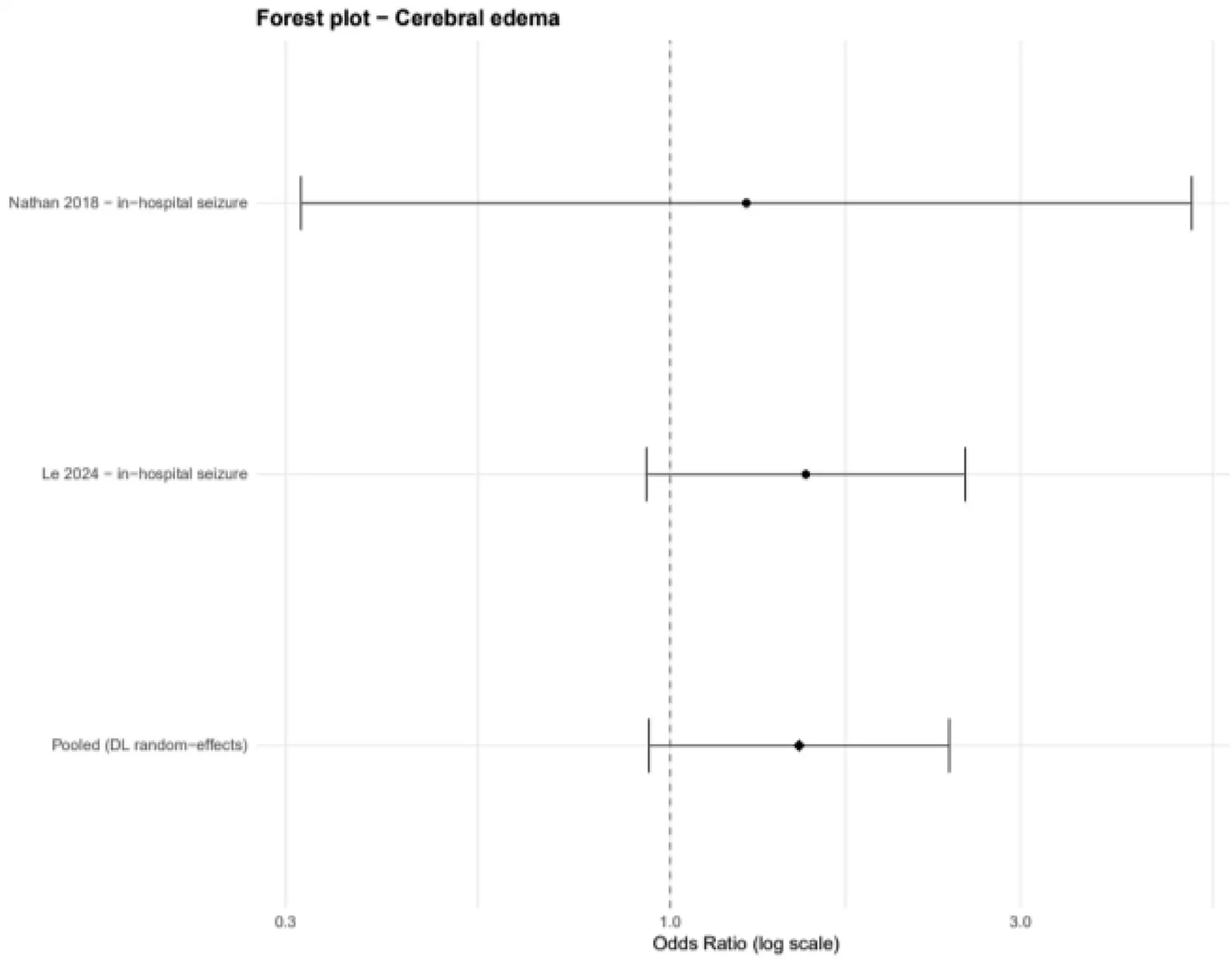
Forest plot for cerebral edema.

**Figure 11:**
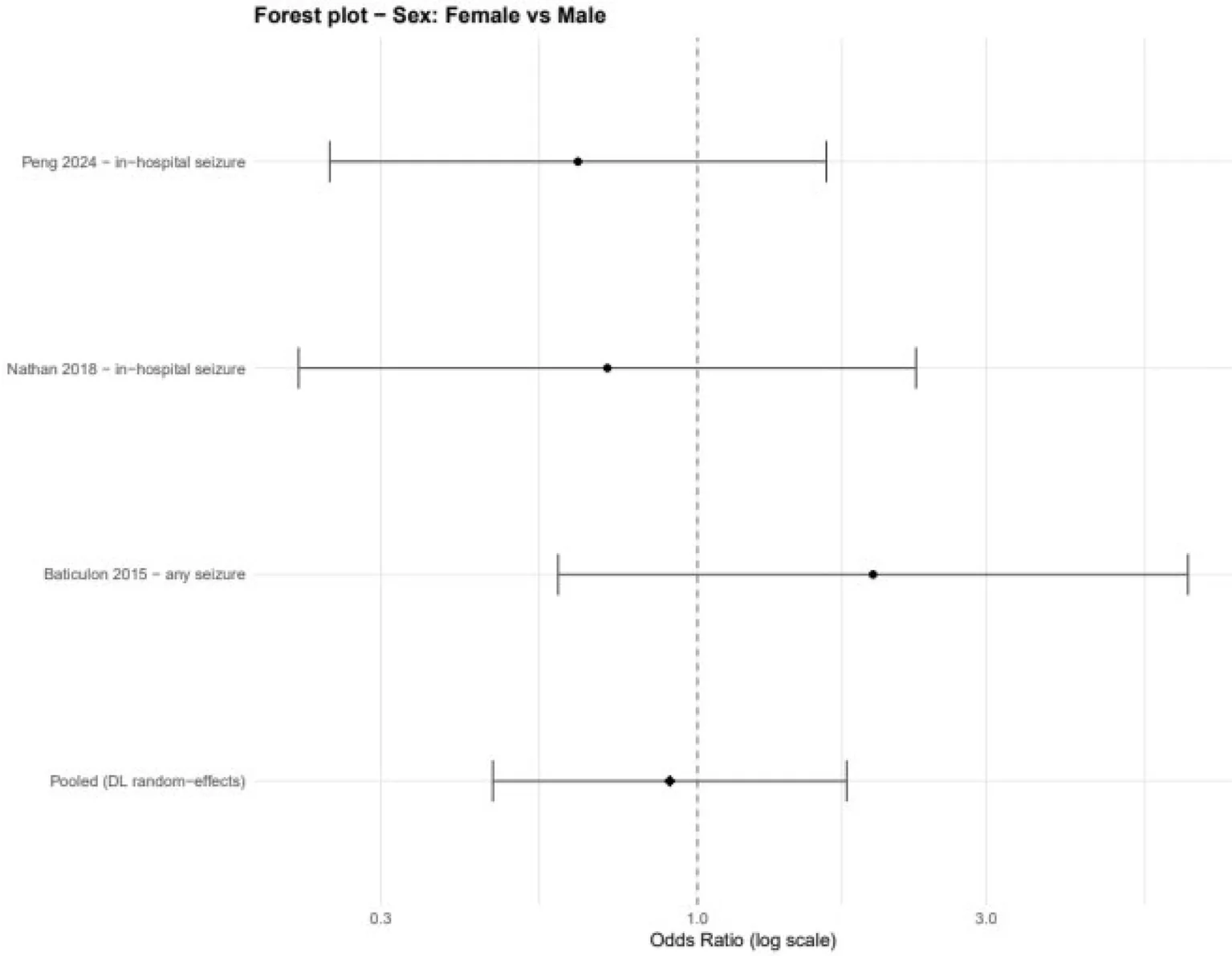
Forest plot for sex (female vs. male).

These factors were not significantly associated with post-aneurysmal subarachnoid hemorrhage seizures. Comorbidity (OR = 2.02, *p* = 0.236) demonstrated moderate heterogeneity (*I*^2^ = 66.9%), while MCA involvement (OR = 1.40, *p* = 0.213), cerebral edema (OR = 1.50, *p* = 0.092), and sex showed no statistically significant associations with post-aneurysmal subarachnoid hemorrhage seizures. These findings should be interpreted cautiously because of the limited number of studies available for several analyses Figures 8–11.

### Publication bias assessment

A funnel plot was generated for the Hunt-Hess 1–3 predictors to visually assess publication bias. The plot had limited diagnostic value because only five studies were available, and no formal Egger’s test was performed, as at least 10 studies are generally recommended for reliable assessment of small-study effects. There was no apparent asymmetry in the visual distribution of the studies but this should be taken with care considering the small sample size.

## Discussion

This systematic review and meta-analysis provide an updated evaluation of factors associated with post-aneurysmal subarachnoid hemorrhage seizures following endovascular aneurysm exclusion. By integrating meta-analytical methods, we quantified the associations between various clinical and radiological factors and post-aneurysmal subarachnoid hemorrhage seizures, providing a more comprehensive quantitative synthesis than previous narrative reviews. By focusing on high-level evidence including studies published through 2024, this review captures contemporary practices and technological advancements in endovascular hardware and neurocritical care. Our primary objective was to evaluate the associations of initial clinical severity, radiological blood distribution, and secondary complications such as hydrocephalus with post-aneurysmal subarachnoid hemorrhage seizures following endovascular treatment Table 4.

**Table 4:** Clinical and radiological predictors of post-aneurysmal seizures.

Predictor category	Specific risk factor	Impact on seizure type
Clinical status (15)	WFNS grade ≥ 4	Increased risk of onset seizures
Imaging (13)	IPH volume > 10 mL	Predictor of late-onset seizures
Age (15)	Age < 51 years	Associated with seizures at onset
Complications (8)	Severe vasospasm	Increased risk of early seizures
Anatomy (15)	Irregular aneurysm sac	Associated with early ictus

### Summary of main findings detailed

This updated systematic review and meta-analysis identified several clinical and radiological factors associated with post-aneurysmal subarachnoid hemorrhage seizures following endovascular aneurysm exclusion. Among the evaluated factors, radiological severity showed the strongest associations with post-aneurysmal subarachnoid hemorrhage seizures, with hydrocephalus demonstrating the highest pooled odds ratio (OR 3.91, 95% CI: 1.22–12.53), followed by Fisher grade 3 and 4 blood distribution (OR 3.29, 95% CI: 1.55–6.97). These findings suggest that greater subarachnoid blood burden and secondary cerebrospinal fluid disturbances are associated with an increased likelihood of post-aneurysmal subarachnoid hemorrhage seizures. However, given the observational nature of the included studies and the limited number of studies available for some analyses, these associations should be interpreted cautiously.

Clinical grading at presentation was also significantly associated with post-aneurysmal subarachnoid hemorrhage seizures. Patients presenting with Hunt-Hess grades 1–3 showed a statistically significant association with post-aneurysmal subarachnoid hemorrhage seizures (OR 3.15, 95% CI: 1.73–5.72), with consistent findings in the in-hospital seizure subgroup. However, this association should be interpreted cautiously given the limited number of included studies and the observational nature of the available evidence (10). While traditional paradigms often focus on high-grade patients, the observed association in patients with Hunt-Hess grades 1–3 may reflect survival bias, with longer survival and greater opportunities for detecting post-aneurysmal subarachnoid hemorrhage seizures among patients with lower initial mortality (9). Conversely, WFNS grades 4–5 remained significantly associated with post-aneurysmal subarachnoid hemorrhage seizures (OR 1.86), although the substantial heterogeneity (*I*^2^ = 60.2%) indicates variability in this association across the included studies. This variability may reflect differences in study populations, perioperative factors, or complications such as vasospasm or delayed cerebral ischemia (8).

Among the factors that were not significantly associated with post-aneurysmal subarachnoid hemorrhage seizures, age demonstrated a borderline association (*p* = 0.066), but substantial heterogeneity (*I*^2^ = 85.1%) indicated considerable variability across the included studies. This variability may reflect differences in study populations, methodologies, seizure outcome definitions, or residual confounding rather than a consistent biological association, and therefore these findings should be interpreted cautiously (8). Anatomical factors such as middle cerebral artery involvement and clinical markers such as cerebral oedema were not significantly associated with post-aneurysmal subarachnoid hemorrhage seizures in this pooled analysis. Although these factors may be associated with seizure occurrence in individual studies or specific clinical settings, the current evidence does not support a consistent association across the included studies. These findings should be interpreted cautiously given the limited number of studies and the observational nature of the available evidence.

### Comparison with prior research

Our results are consistent with and extend findings from classic datasets such as the International Subarachnoid Aneurysm Trial, which established the baseline incidence of post-aneurysmal subarachnoid hemorrhage seizures in endovascularly treated patients. However, our meta-analysis suggests that radiological factors, particularly Fisher grade and hydrocephalus, demonstrated stronger associations with post-aneurysmal subarachnoid hemorrhage seizures than several clinical factors in the available evidence. Previous literature primarily emphasized clinical severity scales as factors associated with post-aneurysmal subarachnoid hemorrhage seizures (8), However, our findings suggest that hydrocephalus (OR 3.91) and dense subarachnoid blood (Fisher grade 3/4) demonstrated the strongest associations with post-aneurysmal subarachnoid hemorrhage seizures. Although these findings are biologically plausible, the underlying mechanisms remain uncertain, and the observed associations should be interpreted cautiously given the observational nature of the included studies.

A strength of this review is the inclusion of recent data from 2022–2024 (e.g., Bögli (10), Nakashima (13)), reflecting contemporary endovascular practice, including the use of newer-generation flow diverters and flow-disruptor devices. While historical series reported post-aneurysmal subarachnoid hemorrhage seizure rates as high as 15.7% for Unruptured aneurysms, the available contemporary evidence does not indicate a clear increase in seizure occurrence with newer endovascular techniques. However, direct comparisons across studies are limited by differences in study design, patient populations, and seizure outcome definitions, and uncertainty regarding factors associated with seizures in patients treated with newer-generation devices remains (8). Notably, the significant association observed for Hunt-Hess grades 1–3 in our analysis differs from earlier meta-analyses, which primarily reported associations between WFNS grades IV–V and post-aneurysmal subarachnoid hemorrhage seizures. One possible explanation is that advances in neurocritical care may increase survival among patients with lower clinical grades, thereby providing greater opportunity for the detection of late-onset seizures or epilepsy. However, this hypothesis remains speculative and cannot be confirmed from the available observational evidence (9, 15).

## Limitations

Despite the significant findings, several methodological limitations should be considered when interpreting these results. The primary limitation is the small number of eligible observational studies (*n* = 6) that provided sufficient quantitative data for meta-analysis, limiting the precision and generalizability of the pooled estimates. This limited the ability to perform robust subgroup analyses, including comparisons between specific endovascular modalities such as flow diversion and simple coiling. Furthermore, the substantial heterogeneity observed for certain factors associated with post-aneurysmal subarachnoid hemorrhage seizures, most notably age (*I*^2^ = 85.1%) and WFNS grade (*I*^2^ = 60.2%), indicates variability in effect estimates across studies. This heterogeneity may reflect differences in patient populations, study protocols, seizure outcome definitions, follow-up durations, and clinical management strategies, including variations in seizure prophylaxis practices (8, 10).

Our findings nevertheless highlight several important clinical and radiological factors associated with post-aneurysmal subarachnoid hemorrhage seizures that contribute to the current understanding of seizure occurrence following endovascular treatment. Radiological severity represented the domain with the strongest observed associations, with hydrocephalus demonstrating the highest pooled association estimate, followed by high-grade subarachnoid blood distribution. These findings are biologically plausible and may reflect the potential contribution of factors such as cerebrospinal fluid disturbances and subarachnoid blood burden to cortical excitability. Clinical grading scales were also associated with seizure occurrence, with Hunt–Hess grades 1–3 showing a significant association with post-aneurysmal subarachnoid hemorrhage seizures, particularly in the in-hospital subgroup, while WFNS grades 4–5 demonstrated a significant association with considerable between-study variability. In contrast, factors such as middle cerebral artery involvement and cerebral oedema were not significantly associated with post-aneurysmal subarachnoid hemorrhage seizures in the pooled analysis, suggesting that their associations may vary across different clinical contexts rather than representing consistent findings across endovascular cohorts.

A significant challenge remains the lack of standardized definitions for seizures following aneurysmal subarachnoid hemorrhage and endovascular treatment across the included studies. Most included studies relied primarily on clinical seizure detection, which may have resulted in underrecognition of subclinical or electrographic seizures detectable through continuous electroencephalography (cEEG). This variability in seizure ascertainment may have contributed to heterogeneity across studies and should be considered when interpreting the pooled findings (16). Moreover, the role of specific endovascular devices (e.g., Woven EndoBridge or intrasaccular flow disruptors) could not be individually assessed because of limited available data. Consequently, the associations between newer endovascular devices, procedural characteristics, and post-aneurysmal subarachnoid hemorrhage seizures remain uncertain.

## Future direction

Future research should prioritize the establishment of prospective, multicenter registries incorporating standardized seizure definitions and continuous EEG (cEEG) monitoring during the acute post-procedural period to improve detection of clinical and electrographic seizures following aneurysmal subarachnoid hemorrhage. Further studies are needed to evaluate the associations between endovascular treatment characteristics, including the use of flow diverters and antiplatelet strategies, and subsequent seizure occurrence. Longitudinal studies with extended follow-up of at least 12–24 months are required to better characterize the temporal patterns of early seizures and late-onset epilepsy after endovascular treatment. Finally, the development of validated risk-assessment models incorporating clinical variables, radiological severity markers such as Fisher grade, hemodynamic parameters, and biomarkers of cortical injury may help refine individualized monitoring strategies and guide future research into seizure management in neurocritical care.

## Conclusion

Higher Fisher grade and hydrocephalus demonstrated the strongest associations with post-aneurysmal subarachnoid hemorrhage seizures following endovascular aneurysm exclusion. Clinical severity scales, including Hunt-Hess grades 1–3 and WFNS grades 4–5, were also associated with post-aneurysmal subarachnoid hemorrhage seizures, although these associations showed greater variability across studies. These findings may support further evaluation of risk-stratified approaches to neurocritical care, including individualized seizure surveillance strategies for patients with greater radiological severity and cerebrospinal fluid disturbances. However, the available evidence does not establish that these factors alone should guide antiepileptic prophylaxis decisions, and further prospective studies are required before definitive changes in clinical practice can be recommended.

## Data Availability

The original contributions presented in the study are included in the article/supplementary material, further inquiries can be directed to the corresponding author.
